# Methodologies for Assessing Disease Tolerance in Pigs

**DOI:** 10.3389/fvets.2018.00329

**Published:** 2019-01-09

**Authors:** Dimitar Nakov, Slavcha Hristov, Branislav Stankovic, Françoise Pol, Ivan Dimitrov, Vlatko Ilieski, Pierre Mormede, Julie Hervé, Elena Terenina, Blandine Lieubeau, Dimitrios K. Papanastasiou, Thomas Bartzanas, Tomas Norton, Deborah Piette, Emanuela Tullo, Ingrid D. E. van Dixhoorn

**Affiliations:** ^1^Faculty of Agricultural Sciences and Food, University Ss. Cyril and Methodius in Skopje, Skopje, Macedonia; ^2^Faculty of Agriculture, University of Belgrade, Belgrade, Serbia; ^3^Agence Nationale de Sécurité Sanitaire (ANSES), Université Bretagne-Loire, Ploufragan, France; ^4^Department of Animal Breeding, Agricultural Institute, Stara Zagora, Bulgaria; ^5^Faculty of Veterinary Medicine, University Ss. Cyril and Methodius in Skopje, Skopje, Macedonia; ^6^GenPhySE, Université de Toulouse, INRA, ENVT, Castanet Tolosan, France; ^7^IECM, INRA, Oniris, Université Bretagne Loire, Nantes, France; ^8^Centre for Research and Technology Hellas, Institute of Bio-Economy and Agri-Technology, Volos, Greece; ^9^M3-BIORES, KU Leuven, Leuven, Belgium; ^10^Department of Environmental Science and Policy, Milan, Italy; ^11^Wageningen UR Livestock Research, Wageningen, Netherlands

**Keywords:** behavior, disease tolerance, environment, performance, stress

## Abstract

Features of intensive farming can seriously threaten pig homeostasis, well-being and productivity. Disease tolerance of an organism is the adaptive ability in preserving homeostasis and at the same time limiting the detrimental impact that infection can inflict on its health and performance without affecting pathogen burden *per se*. While disease resistance (DRs) can be assessed measuring appropriately the pathogen burden within the host, the tolerance cannot be quantified easily. Indeed, it requires the assessment of the changes in performance as well as the changes in pathogen burden. In this paper, special attention is given to criteria required to standardize methodologies for assessing disease tolerance (DT) in respect of infectious diseases in pigs. The concept is applied to different areas of expertise and specific examples are given. The basic physiological mechanisms of DT are reviewed. Disease tolerance pathways, genetics of the tolerance-related traits, stress and disease tolerance, and role of metabolic stress in DT are described. In addition, methodologies based on monitoring of growth and reproductive performance, welfare, emotional affective states, sickness behavior for assessment of disease tolerance, and methodologies based on the relationship between environmental challenges and disease tolerance are considered. Automated Precision Livestock Farming technologies available for monitoring performance, health and welfare-related measures in pig farms, and their limitations regarding DT in pigs are also presented. Since defining standardized methodologies for assessing DT is a serious challenge for biologists, animal scientists and veterinarians, this work should contribute to improvement of health, welfare and production in pigs.

## Implications

Intensively farmed pigs are exposed to a variety of environmental challenges that potentially threaten homeostasis and increase risk for contagious disease outbreaks affecting welfare as well as productivity. Depending on the magnitude of disturbance, the animals are more or less capable of maintaining homeostasis in order to stay healthy, to optimize growth, and to reproduce. There is an imperative to consider in more detail the implementation of alternative approaches to control infectious diseases common in pigs, in addition to the use of therapeutics. One of these strategies for combating pathogens and parasites is the induction of DT. Understanding the relationship between disease tolerance mechanisms and relevant indicators of animal welfare, health, and production might be the key for the reduction of drug usage and for the improvement of animal well-being. Furthermore, understanding DT mechanisms in pigs may lead to better prevention and management of diseases, reducing the cost of treatments and antibiotic use, which in face of antibiotic resistance is essential.

## Introduction

Features of intensive farming can seriously threaten pig homeostasis, causing diseases that are mainly infectious. These are transmissible between pigs and farms, and significantly affect welfare and productivity. To describe how animals cope with these challenges, four related theoretical concepts are used: robustness (Ro), disease resilience (DRe), disease resistance (DRs) and disease tolerance (DT). Breeding for improved Ro, DRe, DRs, and DT has become a major challenge that animal geneticists and veterinarians have to face (1).

In the recent years, there has been more interest in increasing pig tolerance to diseases with a significant economic impact. These diseases, which threaten both pig health and production, are designated as “production diseases,” amongst which infectious and parasitic diseases are of great importance. They are mostly associated with subclinical manifestations (non-specific symptoms of the disease, reduced rate of growth, poor food conversion rate and sporadic rare clinical manifestations) and low mortality, primarily affecting infants (2). When exposed to these diseases, some pigs in the population do not get infected. These individuals obviously possess certain genetic features (1) allowing them to exhibit natural inherent resistance that contributes to staying healthy, even though there are a number of sick individuals living under the same rearing conditions.

Since vaccination and management practices cannot control all common swine diseases outbreaks, there is the need to select animals for DRe and DT traits. Breeding for these traits was found to be more sustainable, economically feasible and desirable, than traditional diseases management practices.

Being resistant for PSSR, for instance with appropriate surveillance programs, such as abattoir health monitoring, would be very appealing for the industry because control measures still remain ineffective (3). Unfortunately, selecting pigs to be more responsive to a specific disease may have some serious drawbacks for their health: this is the case of *Mycoplasma hyorhinis* resistance, indeed, after eight generations the high immune response blood line was more like to develop arthritis (4). Furthermore, addressing the selection to be more responsive to only one kind of bacteria can have unpredictable and detrimental effects when pigs come into contact with other infective agents or pathogens. Thus, prior to the inclusion of these traits in a breeding program, both selection criteria and selection strategy should be carefully evaluated.

Nevertheless, this approach should be deeply investigated since it offers a great opportunity for the pig industry, because this orientation can improve pigs health and welfare, maintaining their productivity and enhancing the public opinion on intensively farmed pigs (5).

For the future, in order to limit damaging behaviors alternative strategies aimed to increase pig Ro [defined by de Goede et al. (6) as relative vulnerability of a system/animal in relation to a specific disturbance] should be kept in mind: early-life conditions, rearing conditions and breeding. Because of its importance, there is an obvious need to extend knowledge about DT and to standardize DT assessment in pigs in relation to genetics and selection, rearing conditions, environmental challenges, stress, behavior and welfare, and pig performance, with special attention to the adoption of automated precision livestock farming (PLF) technologies.

## Concepts and Definitions of Disease Tolerance

Describing standardized methodologies to assess DT in pigs requires a clear distinction between the different concepts of DRs and DT, but also an understanding other concepts such as Ro and DRe. These concepts are characterized by a highly multidimensional nature and the lack of a clear definition and boundaries can lead to confusion (7). The different concepts have been defined as inherent nature of complex adaptive systems and they may all be helpful in describing a variety of ways to assess different aspects of DT in pigs. Although, the concepts have been developed within the fields of ecology and engineering, they are of universal applicability and have context-specific interpretations. They apply at animal or farm level, both complex dynamic systems in themselves. The largest challenge in the application of these concepts lies in finding practical performance indicators.

DRs is the ability to actively diminish the pathogen burden or prevalence through the inhibition of the infection and through the reduction of bacteria/viruses growth rate. Also, the level of control that the host can have over the pathogen lifecycle falls within DR definition (8). DT definition, in contrast, relies on the host's ability to mitigate the detrimental pathogen impacts limiting the possible damages (5) or the net impact on performance of a given level of infection (8). It has been described more than 50 years ago looking at the ability of highly infected plants to survive while limiting tissue damage (9). The first evidence of tolerance against infectious diseases in mammals was next provided by Råberg et al. (10). It is considered that DT relies on the tissue damage control, a series of protective mechanisms, based on cellular and adaptive response of the organism that the host implements to protect parenchyma tissues from stress, dysfunction and/or damage (11). Also the host's capability to reduce the effect of an infection on its fitness can be considered as DT. DT action can be direct, reducing the pathogen damage (direct tolerance) indirect, reducing damages caused by the immune response (indirect tolerance). The main difference between DRs and DT is interaction, or lack of interaction between host and pathogen. DT mechanisms do not directly affect the pathogen, although a distinction between the two mechanisms is not always clear (5); DRe is then seen as a related concept describing the capacity to change in order to stay healthy and often defined as the productivity in the face of an infection, or as the capacity to bounce back to normal functioning after perturbation. If systemic resilience decreases, risk of morbidity and mortality increases (12).

Instead of the current WHO definition in which health refers to a state of complete physical, mental, and social well-being and not merely to the absence of disease or infirmity, it has been discussed to reformulate this theoretical concept into a more dynamic definition, *i.e.*, “the ability to adapt and to self-manage” (13). This new concept which defines health as the ability to adapt implicate that DT appears as an important feature.

The definitions, diagrammatically presented by Bishop (14), give directions toward possible measures to assess DT, DRs, or DRe at group level. According to Richardson (15), DRe is the recovery capability of an individual from illness; both DT and DRe strictly depending on host and pathogen genetics can influence the healing process. It should be borne in mind that DT mechanisms can prevent tissue damage or can improve tissue function, without interfering with pathogen load, thus reducing the severity of the effects of the disease (11).

Other related concepts for assessing features of complex adaptive systems facing perturbations are stability and vulnerability, as well as Ro and DRe which were described and visualized by Urruty et al. (7). Both concepts of stability and vulnerability could be used when assessing DT. Stability refers to fluctuations in signals assessed from the studied system and indicates how much it is affected by the perturbation. Vulnerability indicates how much the animal is potentially affected by infection, but does not necessarily describe the degree of recovery after infection. With regard to Ro, two forms can be distinguished; the passive and active Ro: (i) DRs, i.e., the withstanding or tolerance of perturbations, and (ii) flexibility, i.e., the ability to adapt the configuration of the system in order to limit damage (16). Thus, resistance and tolerance are both seen as aspects of the Ro, whereas the active Ro closely relates to the resilience concept. In the context of diseases, the concept of DT seems to be the most complete and suitable concept to be used.

To describe standardized methodologies for assessing DT in pigs, the following aspects with regards to the study should first be defined: what kind of system is studied (pigs, at individual, group or breed level), what kind of output is targeted (behavior, production, reproduction or sickness) and against what kind of perturbation (specific diseases or other specific or generic perturbations) (7). To date, primary mechanisms of tolerance are still largely unknown, although they rely on tissue repair mechanisms and tight regulation of immune responses to prevent host tissue damage (17, 18). Especially the emerging role of integrative, systems-oriented approaches to understanding the complex mechanisms underlying infection, immune response and inflammation, leads to revealing more and more insights in system dynamics and cause effect-relationships in the primary crucial immunological mechanisms of tolerance (19–21). For example, the concept of protective tolerance toward fungal infections was described in mice and humans and was shown to depend on IL-10 secreted by regulatory T cells (20). New approaches to the mathematical modeling of host-pathogen inter-actions, when further adapted to the specific biological context will be of future additional value when assessing DT. Tolerance can be identified as the regression of the host performance vs. the infection intensity, and, the steeper the slope the lower the tolerance (22). The adaptive response, which is also referred to as adaptation, relies on the activation of several evolutionarily conserved genetic programs that confer protection against these agonists. Some of these genetic programs overlap with those regulating stress and damage responses conferring tissue damage control and DT to infections (17).

Difference in tolerance between individuals might be related to genetic variation, environmental or other external factors or combinations of these. Deterioration in climatic conditions and air quality inside a pig house can trigger adverse effects in pigs, impairing their welfare, health, and growth. Guy et al. (23) highlighted how common environmental challenges in pig production, such as temperature and poor air quality, may be included in models used to investigate the mechanisms of DRs and DT.

Genetic variation in tolerance implies that genotypes differ in their response to pathogen burden, which basically represents a genotype by environment interaction (24). The response of a genotype to varying environmental conditions identifies the genetic variations in DT. However, Bishop (14) pointed out that while geneticists believe they are measuring tolerance they are often looking at a composite trait combining DT and DRs. This aspect was further explored by Doeschl-Wilson et al. (25), who provided a mathematical framework to quantify a better measure of tolerance for an individual based on within-host pathogen burden.

**Figure 1:**
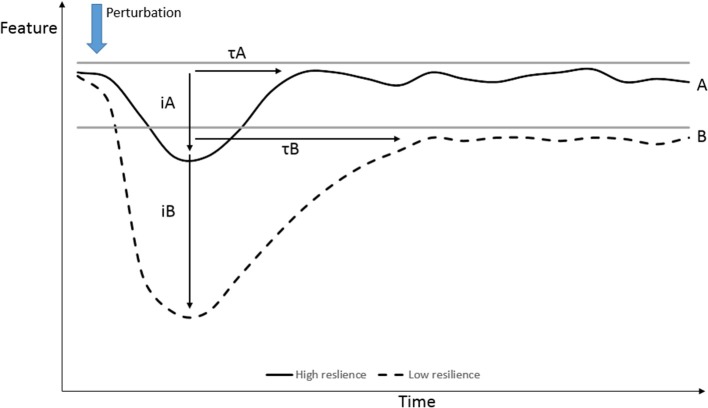
Quantification of performance responses after perturbations, resistance (iA and iB), recovery or halftime (τA and τB) to (new) equilibrium and robustness (equilibrium level after perturbation). [Modification Urruty et al. (7)].

A variety of targeted outputs or performance indicators [as shown on the y-axis by Bishop (14)] could be defined. The performance can be more generic and more related to production at individual or group level (e.g., growth or reproductive performance) or to the adaptive responses to absorb disturbances at individual level (e.g., physiological reactions, behavioral reactions, sickness behavior). The responses after perturbations can be quantified by different aspects of the response as shown in Figure 1 by means of level (A and B), impact of disturbance (iA and iB) or half time to return to equilibrium (τA and τB), or by other dynamical characteristics of the signal that was measured (such as variance, temporal autocorrelation) and are referred to as dynamical indicators of resilience (12). The variable to be measured can be directly (e.g., immunological) or indirectly (e.g., growth) related to underlying mechanisms. The choice of performance variable is dependent on the expected impact of disturbance and, in the case of DT, on the expected impact of the specific disease.

Differences in viral load and viral clearance after infection, as described by van Dixhoorn et al. (26), could be used as an example of the quantification of possible performance responses after infection. The peak of virus amount in the blood (at day 4) represents the resistance, which was similar for both groups, whereas the speed to reduce the virus amount represents the capacity to react to the infection. This faster virus clearance also led to reduced histo-pathological Porcine Reproductive and Respiratory Syndrome Virus (PRRSV)-associated tissue damage in the lungs.

## Conceptual Framework For Assessing Disease Tolerance in Pigs

It is of crucial importance to standardize methodologies for the assessment of DT in pigs. In doing so, comprehensive consideration should be given to the relationships between the stability, vulnerability, avoidance, DRs, DRe, DT, and Ro of the pig as described above, type of illness, breed, gender, age, living conditions, phenotypic properties, genetics and selection, behavior, stress and welfare, productive and reproductive traits. Since it is difficult to distinguish DT from DRs when health status or pathogen load are measured alone, assessing DT would benefit from the combined measurement of animal health parameters, pathogen burden, and distribution in the body (22, 27). If possible, combined measurements of disease parameters and pathogen load over time would help to map disease trajectories.

To be standardized, a methodology has to be similar to a unique described model: a standard. However, diseases in pigs are diverse, with multiple DT mechanisms. These mechanisms tend to prevent, reduce, or counter the pathological alterations caused by infections in order to preserve host fitness. They can be specific, related to the causative agent pathogenicity, such as changes in blood pressure or local tissue hypoxia, but can also be less specific, related to stress, tissue damage repair or homeostasis maintenance, such as body weight or body temperature. Thus, criteria to assess DT through health parameters are multiple, according to the pathogen and its target organ. If the measures have to be standardized, they have to focus on several and non-specific mechanisms and assess physiological parameters or behaviors which are involved. If the measures are made in a context of reaction to a specific pathogen, they can be targeted to specific mechanisms and biomarkers.

For the methodology to be reliable and valid, the measurements themselves have to be reliable and conform to the imperatives for method validation in general. The measures, for either health parameters or pathogen burden, have to be repeatable, sufficiently sensitive and specific. For health parameters, this means that each of them can be benchmarked to the standard that indicates non-infected and healthy pigs. If assessment requires different steps, including recording or sampling in the animal and then laboratory manipulations and/or mathematical modeling, each step has to be validated. If an operator performs the measure, it has to be robust with a low inter-operator variation. In the Welfare Quality protocols, for example, inter-observer and test-retest reliability is not perfect for each criterion (28, 29).

As DT is subject to individual variation (17, 30), the number of animals to be sampled has to be a reliable representation of the batch. As many measurements are done on farms, DT assessment has to be done in a relatively short period of time and should be easy to perform under commercial conditions and in various housing designs. Finally, in the context of commercial production, the assessment should not be too expensive. For this, also automated sensor monitoring seems to be of additional value.

Very important parameters for assessing pig DT are the expression of all normal behavioral systems, body condition, constitution and health status, susceptibility to stress, productive, and reproductive traits. All these traits should be monitored through several generations of pigs, strictly assessed and excluding those that are subjected to any undesirable deviations. Furthermore, any potential interactions between genotypes and the environment should be considered, as well as the feature variables of the disease.

Health status can be monitored through the assessment of physiological parameter such as body temperature, oxygen, pH, osmolarity, or glucose concentrations (17). These physiological parameters are not specific but they can be used as indicators of DT when compared to data relative to pathogen load. However, in animal breeding, those traits are not easy to measure and quantify. Some routinely collected data that are used as indirect measure, enabling the modeling and the quantification of resistance and tolerance, and allowing the assessment of the benefits of selection, and identifying which traits (DRs, DT, or both) should be object of selection (23).

Because of the inter-relationship between tissue damage control in DT and immune-driven host mechanisms in DRs, assessing immunocompetence, through measurement of innate and adaptive immune traits, is another way to evaluate DT. Knowledge in porcine immunology has deeply extended during the last decade, allowing the development of relevant tools for porcine immunology (31). Serum acute phase proteins, such as haptoglobin, C-reactive protein and pig-MAP, were described as good indicators of pig health (32). Other adaptative and innate immunity markers, such as blood IL-1α and TNFα levels were described in pigs, but further studies are required to assess their relevance as health biomarkers in pigs (33).

**Figure 2:**
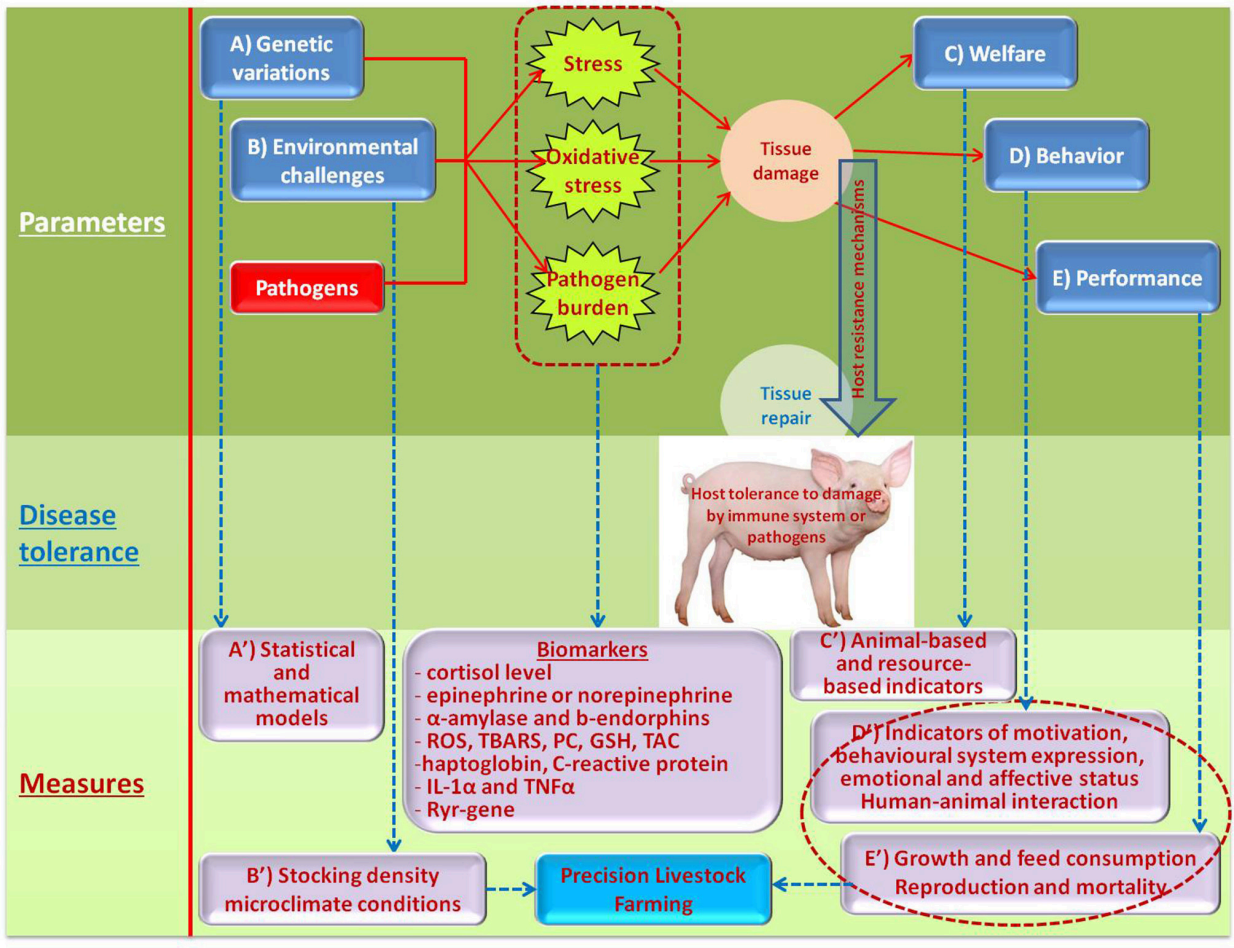
Illustration of conceptual framework for assessing DT in pigs.

A variety of target inputs that can inflict the host resistance mechanisms and possible measurements to assess DT are shown on Figure 2.

Assessment of pathogen load and distribution is not easily done in practice, especially when pathogens are “hidden” in tissues that cannot be sampled on live animals. However, for few pathogens such as PPRSV viral load quantification is possible on blood samples (34), while tools are still lacking for most of the pathogens encountered in pig farms.

### Genetic Biomarkers for Disease Tolerance

Genetic analysis methods for quantifying individual host response to infectious pathogens were described by Hermesch et al. (24). The genetic analysis of tolerance to infections was reviewed in detail by Kause and Ødegård (35) that identified three statistical methods useful to investigate genetics of DT-related traits: random regressions, the cure model for time-until-death data and the normal mixture model. The molecular pathways underlying host DRs and DT to pathogens were explained in detail in the paper by Glass (36). Several effects can occur due to variation in DT, such as genotype re-ranking and changes in genetic and phenotypic variation in host performance along the pathogen burden trajectory, contributing to environment-dependent genetic responses to selection. Such genotype-by-environment interactions can be quantified by combining random regressions and covariance functions. To apply random regressions, pathogen burden of individuals needs to be recorded (37, 38). When an organism owns DT traits, its performance change according to the pathogen burden, hence applying random regression models allow estimating genetic parameters and breeding values for tolerance (37). Thus, to evaluate DT, host performance can be regressed against pathogen burden of individuals. Furthermore, DT genes and pathways play a role in reducing immunopathology or enhancing the host's ability to protect against pathogen associated toxins (Figure 2, A). Also, candidate tolerance genes may include cytosolic pattern recognition receptors (PRRs) and unidentified sensors of pathogen growth, perturbation of host metabolism and intrinsic danger or damage-associated molecules. In addition, genes are controlling regulatory pathways and tissue repair to tolerance candidates. The identities of distinct genetic loci for DRs and DT to infectious pathogens in livestock species remain to be determined. Obtaining the tolerance phenotype through group estimates as the first step toward genetic selection for host tolerance to infectious pathogens was considered by Doeschl-Wilson et al. (39). A mathematical model to study the resistance and tolerance to infection at the animal and population levels was described by Detilleux (40). Discussion of breeding for Dre or DT in selection of pigs for improved coping with health and environmental challenges can be found in the paper by Guy et al. (23).

**Genetic biomarkers for disease tolerance**

**Challenge:**

- quantifying individual host response to infectious pathogens
- genetic analysis of tolerance to infections
- environment-dependent genetic responses to selection

**Variable:**

Pathogen burden and host performance of individuals

**Key point:**

To evaluate DT, host performance can be regressed against pathogen burden of individuals. Several strategies can be used to genetically manage diseases, such as breed substitution, cross-breeding and within-breed selection.

If standardized methodologies for assessing DT in pigs start from the fact that, within a population, different individuals have different genetic traits related to DT, then for further breeding the most suitable ones should be chosen. Several strategies can be used to genetically manage diseases, such as breed substitution, cross-breeding and within-breed selection. The adoption of a strategy depends on the specific disease, the production environment and the resources available. For example, only if genetic markers associated to desired traits, it is possible to perform within-breed selection. Selecting pigs for Ro might be an opportunity to select for DT. Individual variations in Ro were shown to rely upon hypothalamo-pituitary-adrenal (HPA) axis activity (41, 42). High HPA axis activity was shown to increase immune capacity, so selecting pigs for this trait could increase their resistance to pathogens (43).

### Environmental Challenges and Disease Tolerance

Deterioration in climatic conditions and air quality inside a pig house can trigger adverse effects in pigs, impairing their welfare, health and growth (Figure 2, B). Guy et al. (23) emphasized the importance of environmental parameters in models used to investigate DRs and DT mechanisms. These parameters are common in intensive pig farming and include external stressors such as extremes of temperature and poor air quality. Martínez-Miró et al. (44) stated that environmental parameters such as temperature, humidity, light, concentration of dust and gases, ammonia levels and sound intensity constitute serious environmental stress to pigs. They also underlined that pigs' thermal comfort is not linked only to ambient temperature, but on the effective temperature, that is the sum of factors as ambient temperature, ventilation, floor type and bedding material, among other factors. Supporting this, Banhazi and Rutley (45) mentioned that housing and management factors (e.g., ventilation control, stocking density, stocking rate) affect the thermal environment in pig houses. Choi et al. (46) found that harsh environmental conditions (temperature, humidity, and high concentration of harmful gas, including ammonia,) negatively affect productivity, physiological, and behavioral status of growing pigs. In particular, adrenocorticotropic hormone, stress, posture, and eating habits were all affected by the environmental parameters. For example, lying and excreting behaviors of growing pigs were greatly affected by high temperatures, while humidity has a minor effect, as reported by Huynh et al. (47). To assess the welfare of pigs, they also suggested that the number of pigs lying on slatted floor gives an indication of high ambient temperatures. Pedersen et al. (48) mentioned that high dust concentrations have been related to reduced growth rate increased respiratory health problems (i.e., lung damage, pneumonia, atrophic rhinitis) and disorders of the immune system in pigs. Lee et al. (49) evaluated the effects of “clean” and “dirty” environmental conditions on the growth and on the responses of endocrine system of male weaned pigs in. In that study, they reported the negative effect of a “dirty” environment on the feed intake, growth rates and IGF-I, cortisol and β-endorphin concentration compared to pigs housed in a “clean” environment. Also heat stress has a negative effect, as reported by Lee et al. (50) on the permeability, oxidative stress, and inflammatory responses in the gut. Cui et al. (51) investigated the proteomic response of the liver of finishing pigs to the same stressor, founding that chronic heat stress alters the expression of hepatic proteins, which are the regulatory mechanism of oxidative stress, redox state, and apoptosis. Qu et al. (52) found that heat stress in pigs may favor increased triglyceride storage due to adipose tissue–specific responses. The study of Alarcon et al. (53) provides evidence of the association between environmental and management factors and the severity of post-weaning multi-systemic wasting syndrome. Additionally, the authors cited some papers that concluded that environmental stressors and overstocking are factors that may be linked to the development of this disease and to negative welfare effects in general. To conclude, there are many environmental challenges that can act synergistically and influence a number of systems, causing reactions which can affect DT.

**Environmental Challenges and Disease Tolerance**

**Challenge:**

Stocking density, heat stress, poor air quality, deterioration in microclimatic conditions

**Variable:**

Lying behavior, excreting behavior, growth, feed intake and health

**Key point:**

Stressors related to environmental (e.g. microclimate inside the barn, air quality) and housing (e.g. ventilation, stocking density / rate) conditions should be effectively managed in order to ensure that their impacts on DR and DT will be minimized.

### Stress and Disease Tolerance

Stress is the generic term used to describe non-specific responses of the body to all kinds of challenges (social, environmental, metabolic, immunological, etc.) that threaten homeostasis (44, 54). Thus, any decrease in stress exposure is expected to increase both health and welfare of individuals. For example, housing pigs in stressless conditions was shown to decrease viral load and tissue damages in case of co-infection by PRRSV and *Actinobacillus pleuropneumoniae* (26). The response to stress is not a reflex but results from complex interactions between each individual and its environment (55) and may reflect DT. It involves the activation of two main pathways: the HPA axis and the sympathetic-adreno-medullary (SAM) system, leading to the respective release of glucocorticoid and catecholamine [epinephrine (E) and norepinephrine (NE)] in the periphery. Besides, stressors also activate other central structures such as the amygdala, that controls behavioral response to stress, and the loecus coeruleus (LC), that release central NE to shape the stress response. The LC-NE activity can be down-regulated by endogenous opioids, such as β-endorphins, that act as “anti-stress” mediators (56). Hence, individual variations in any contributor of the stress response can determine stress resilience or vulnerability and thus affect DT. HPA axis activity can be assessed through cortisol level measurement in different types of samples. Serum or plasma cortisol level is the most widely used despite strong nycthemeral variability. It tends to be replaced by urinary or salivary cortisol concentration determination with the additional advantage of resulting from non-invasive sampling. Cortisol accumulation in pig bristles is sometimes used and reflects cumulative HPA axis activity (57). SAM activation can be evaluated through plasma E and NE concentrations, but it is also subjected to diurnal variations. Measuring salivary α-amylase activity is another approach to assess SAM activation (58). Also, salivary chromogranine A (CgA) level is a reliable marker of SAM activation in pigs (59), since it is released in saliva in response to catecholamine while being not affected by age, gender, or circadian rhythm. Finally, few studies evaluated the potential use of plasma β-endorphins level to monitor individual stress response (60).

Until now, research programs had mainly focused on the description of the deleterious impact of stress mediators on immune responses. However, recent data suggest that stress mediators could also promote tissue repair and increase DT, especially through their action on tissue-resident macrophages (61, 62). In this context, further studies are warranted to evaluate the predictive value of stress mediators for DT assessment.

**Stress and disease tolerance**

**Challenge:**

Social, environmental, metabolic, immunological stresses

**Variable:**

Serum or plasma cortisol level, plasma epinephrine and norepinephrine concentrations

**Key point:**

Minimize internal and external stressors

### Oxidative Stress in Disease Tolerance

Oxidative stress can be defined as an imbalance between free radical production and opposing antioxidant defenses. There are growing indications that oxidative stress significantly damages organ function and plays a major role in the etiology and pathogenesis of several metabolic and infectious diseases in animals. Oxidative stress is known to be associated with variety of neurodegenerative diseases, metabolic syndrome, atherosclerosis and carcinogenic process (63). Virus-induced oxidative stress has been reported during HIV, influenza virus, HBV, hepatitis C virus, encephalomyocarditis virus (EMCV), respiratory syncytial virus (RSV), dengue virus (DENV) (64). Disturbances in the normal redox potential of aerobic cells can cause toxic effects through the production of oxygen-derived free radicals (ROS) that induce destruction of macromolecules and damage vital functions of the cell. As result, the animal can manifest alterations of physiology and behavior and poor growth performance, and suffer from various diseases.

One component of the local and systemic host defense mechanisms is inflammation. These non-specific responses contribute to the innate immune system's ability to neutralize invading pathogens. However, inflammation has adverse effects on the organism, and there is growing recognition of the harmful effects of excessive or chronic inflammation for the animal, especially with respect to its metabolic function and their role in tissue damage. Although numerous factors can initiate inflammation, the excessive accumulation of ROS plays a central role in mediating uncontrolled inflammatory responses and stress-adaptive responses to tissue damage control (11). Therefore, oxidative stress is an underlying cause of depressed immune system function exposing the animals to health disorders.

**Oxidative stress and disease tolerance**

**Challenge:**

Imbalance between free radical production and opposing antioxidant defenses

**Variable:**

Behavior, growth, health

**Key point:**

Future research should be oriented to the identification of a reference panel of biomarkers of oxidative stress to be used as tools for assessment of disease tolerance.

Quantifying the oxidative stress gives the measure of physiological defenses real status and the capacity in preventing the occurrence of correlated pathologies. Measuring oxidative stress is not easy, and it cannot be performed with common methods of analysis. Oxidative stress can be monitored with several biomarkers (anti- and pro-oxidants) that can be assessed in plasma and/or erythrocytesurine and saliva. The capacity of antioxidants in reducing the harmful impact of accumulated ROS on innate immunity of host tissue can be used for assessing the DT in animals. The most appropriate measurements for determination of the redox status of the organism are: stability of the cell membrane, direct measurement of ROS concentration in plasma or serum, total antioxidant activity, measurements of antioxidant enzyme activity, measurement of non-enzymatic antioxidant concentration, measurement of glutathione level and measurement of products of lipid, protein, and DNA oxidation (65). The most used non-invasive measurements of oxidative stress in blood samples for assessing tissue damaging control as underline factor for DT are: xanthine oxidase, thiobarbituric acid-reactive substances (TBARS), protein carbonyls (PC), reduced glutathione (GSH), oxidized glutathione (GSSG), catalase, and total antioxidant capacity (TAC) (66). In this context, future research efforts should be oriented to the identification of a reference panel of biomarkers of oxidative stress to be used as tools for assessment of DT. Combination of oxidants and antioxidant markers can provides a reliable indication about the redox status and DT in the organisms.

### Welfare, Normal Behavior, Emotions, and Sickness Behavior

It seems crucial to develop practical methodologies for assessing DT in pigs based on the expression of normal behavioral systems, emotional affective states, and sickness behavior (Figure 2, D). If an assessment of the main behavioral systems (reactivity, exploration, kinetic behavior, ingestion, social interaction, reproduction, territoriality, defecation and urination, rest and sleep) documents that the individual shows the appropriate nature, patterns and strategies of the behaviors, which are well-expressed, uninhibited and without disorder, it certainly has a predisposition to express good DT. Well-expressed behavioral systems, together with affective or emotional state, and the absence of sickness behavior are essential components of overall health, as well as the state of well-being and the welfare of pigs (67, 68). Assessments of the behavioral systems, emotional state and sickness behavior of pigs have to be carried out daily and systematically (68).

Interpreting and study animals' emotions is a demanding task, but pigs express clearly their emotions when they exhibit their behavioral repertoire (play, fear, and stress responses), and their sensitivity to the emotions of their counterparts (69). Essentially, emotions motivate an animal's behavior. When studying how certain management and production systems impact animal affective states, researchers, veterinarians and producers usually focus on the negative emotions. For example, farmers try to improve any practice that causes fear, such as mixing, transport, and handling. Frustration is another emotion that is well-studied and often manifests itself in the expression of abnormal behaviors. For example, pigs are highly motivated to perform certain behaviors such as rooting and, when they are prevented from doing so, they may begin to develop oral stereotypes (68).

Ethologists have designed a variety of experimental approaches that can be used to determine how animals perceive various housing conditions and management systems. Preference tests can be used to measure an animal's motivation for resources or environments with the underlying assumption that animals approach what they find positive and avoid what they find aversive (67).

Monitoring and assessing pig welfare provide the farmer with methods for benchmarking the state of welfare (Figure 2, C). These benchmarks can be used for decision making regarding the best management practices and provide a way for producers to demonstrate that their pigs are receiving a certain level of care. On-farm measures of animal welfare typically fall into two categories: resource-based or animal-based measures. Details about assessment of resource-based and animal-based measures can be found in the Welfare Quality® (70) assessment protocol for pigs (sows and piglets, growing and finishing pigs). In addition, the behavior and health of pigs from birth to slaughter should be permanently kept under surveillance.

To correctly draw the welfare status of pigs, as well as their pain or suffering experience, the best way is the combination of the examination clinical aspects and the knowledge of the normal behavioral repertoire. Knowledge of temperament and affective states would be helpful. For the purposes of assessing DT, however, additional animal welfare developments and drivers [see Welfare Quality® (70)] such as damaging behavior, temperament and affective states, need to be taken into account. Therefore, assessment of negative feelings and positive experiences need to be addressed precisely, and the concepts of coping style, personality and temperament need further study (68).

It is well-known that during acute stages of disease (i.e., sickness behavior) pigs modify their behavior reducing their movements, feeding, and drinking and interacting less, and at the same time they tend to huddling, shivering and resting more. The expression of behavior during disease is context-dependent, affecting the likelihood of clinical signs being expressed in certain social environments. Pigs sometimes present only subtle behavioral indicators of disease and pain and are viewed as “stoic” due to their evolutionary niche (71).

Benefits of the sickness behavior not only positively affects the host resistance, but Medzhitov et al. (22) suggest that it promote host tolerance to infection. Indeed, behaviors typical of sickness, such as anorexia and fatigue may be involved in preserving vital processes and promoting stress tolerance in multiple tissues. Indeed, anorexia has been shown to enhance tolerance to *Salmonella* infection in flies, while at the same time it reduces resistance to *Listeria* infection (72), meaning that presence or absence of protective effects of sickness behavior and their mechanisms are pathogen-specific.

**Welfare, normal behavior, emotions and sickness behavior**

**Challenge:**

Mixing, transport and handling, any stressful situation

**Variable:**

Behavior (reactivity, exploration, kinetic behavior, ingestion, social interaction, reproduction, territoriality, defecation and urination, rest and sleep), growth, health

**Key point:**

Monitoring and assessing pig welfare provide the farmer with methods for benchmarking the state of welfare to decide accordingly the best management practices.

### Performance

As pigs have been subjected to long-term selection on their productivity, and more recently on their Ro (24), the monitoring of their performance to assess DT may not be the most sensitive parameter. Indeed, robust animals tend to better cope with environmental stressors such as pathogens. For example, pigs tolerant to PRRSV showed a normal weight gain in despite the relatively high virus load (73). However, poor growth or reproductive performance is animal-based indicators that can be used to assess health and welfare of the animals (Figure 2, E). Analyzing the operation's veterinary records and past diagnostic laboratory reports provides a picture of previous areas of concern and guidance on the expected health status of the herd. Production sites should have treatment and mortality records on-site. These records are helpful in determining the total number of pigs in the original batch, the number of mortalities, and the chronology of mortalities to date. A good practice is to record a presumed death reason and to educate producers on how to properly evaluate and record mortalities; this aids an understanding the relationship of different production parameters and DT (2). In experimental conditions, these parameters can be regularly recorded and monitored and each deviation can be used as a warning signal. However, this supposes that they can be measured regularly with accuracy and that they are benchmarked to a standard.

Under farming conditions, animal measures are seldom recorded, often only at a few different points in the animal's life. For example, pig body weight can be measured at weaning, at the beginning and at the end of fattening, when the pigs are slaughtered. Other measures, such as back fat thickness can be easier to perform on the farm, since animals do not need to be moved to fixed facilities. Reproductive performance measures of sows are easier to assess, since no specific equipment is needed.

However, with the advent of Precision Livestock Farming (PLF) technology, many measures should become easier and faster to monitor on farm at the individual level and this will provide new opportunities to assess DT. For example, data related to feed consumption and feed efficacy could be analyzed in real time and each deviation highlighted for attention. However, standard growth and reproductive performance parameters have to be known for each genetic line in order to appreciate any deviation, even if narrow.

**Performance**

**Challenge:**

Understanding the relationship between different production parameters and DT

**Variable:**

Pig body weight, back fat thickness, feed consumption, feed efficacy, reproductive parameters

**Key point:**

Production sites should have treatment and mortality records on-site. Animal performance measures should be recorded, which should become easier with the advent of PLF.

### Automated Precision Livestock Farming Technologies and Their Limitations

DT can be quantified through physiological variables, such as health status or performance, as well as through behavioral variables related to welfare. In the past few decades, a lot of sensor technology has been developed to monitor measures of health status, performance, behavior and welfare fully automatically 24 h a day. Two-dimensional camera technologies have been used to monitor activity, drinking behavior and feeding behavior (74). Three-dimensional video recordings have been applied in the automated detection of aggressive events in pens of group-housed pigs (47, 75). Recently, research has been conducted to automatically monitor the skin temperature of pigs using infrared cameras in order to detect injuries and infections (76). The big advantage of these systems is that they are relatively cheap and that one sensor can be used to monitor different pigs. The main drawback of these systems, however, is that they are intended to measure variables at a group level. Given the definition, we should be able to monitor individual animals if we want to measure DT adequately.

In recent years, researchers have made efforts to develop systems that can monitor pigs individually. Maselyne et al. (77, 78) measured drinking and feeding behavior in individual pigs by using radiofrequency identification (RFID) ear tags. The difficulty in this approach is that it requires at least one sensor per pig, which translates into additional costs and labor when tagging the pigs. Work is being done to develop camera systems that can track individual pigs, but their performance is not yet good enough for the market (79). To this date, RFID tagging is the most reliable way to track the performance and behavior of individual pigs, and accordingly to monitor DT in individual pigs.

**Automated Precision Livestock Farming technologies and their limitations**

**Challenge:**

Monitor individual animals automatically and continuously to measure and manage DT

**Variable:**

Sensor technology to measure the health, welfare and behavior of pigs, as well as their environment

**Key point:**

There is an abundance of sensors on the market to monitor the environment and the pigs automatically and continuously. However, algorithms for camera technologies to track individual pigs are not on point yet, and the reference method of RFID tagging can be costly.

If we want to use physiological and behavioral measures to monitor DT, it is important that we consider all factors that have an influence on these measures. For instance, growth will not only be affected by DT, but is also altered through environmental conditions such as temperature and humidity. One way to take these factors into account is to keep them as constant as possible, which requires monitoring and managing the environment. Another method consists of only monitoring the environment continuously and taking the changes in environment into account statistically when assessing DT. Either method requires that the barn be equipped with sensors to monitor the environment continuously. To date there is an abundance of affordable environmental sensors on the market, which means that it is possible to control for a series of factors that affect the physiological variables that are used to quantify DT.

## Conclusions

Assessing DT mechanisms that limit disease severity without affecting the pathogen load, by protecting the infected host from tissue damage, is an issue that deserves increased focus. In order to support this concept of innate defense, it is necessary to understand its complex nature and the factors on which it depends. It is necessary for all the elements on which DT depends to integrate into one comprehensive and standardized methodology. It is important to include methodologies based on monitoring of growth and reproductive performance, welfare, emotional affective states, and sickness behavior for the assessment of DT, and to utilize methodologies based on the relationship between environmental challenges and disease tolerance. Automated Precision Livestock Farming technologies available for monitoring performance, health and welfare-related measures in pig farms should be developed according to these principles and their limitations addressed in further research. Methodologies for assessing DT should contribute to improve health, welfare and production in pigs. In addition, breeding for DT in pigs should be part of an integrated health herd program and should be applied to the entire industry with appropriate surveillance programs, such as abattoir health monitoring. Finally, a winning strategy to control diseases and prevent production losses could be to selection of animals that are simultaneously resistant and tolerant.

## Author Contributions

All authors listed have made a substantial, direct and intellectual contribution to the work, and approved it for publication.

### Conflict of Interest Statement

The authors declare that the research was conducted in the absence of any commercial or financial relationships that could be construed as a potential conflict of interest. The reviewer FM declared a shared affiliation, with no collaboration, with several of the authors, JH and BL, to the handling editor at the time of review.
